# Neuroscience of Childhood Poverty: Evidence of Impacts and Mechanisms as Vehicles of Dialog With Ethics

**DOI:** 10.3389/fpsyg.2017.00061

**Published:** 2017-01-26

**Authors:** Sebastián J. Lipina, Kathinka Evers

**Affiliations:** ^1^Unidad de Neurobiología Aplicada (Centro de Educación Médica e Investigaciones Clínicas “Norberto Quirno”-Consejo Nacional de Investigaciones Científicas y Técnicas)Buenos Aires, Argentina; ^2^Centre for Research Ethics and Bioethics (CRB), Uppsala UniversitetUppsala, Sweden

**Keywords:** interdisciplinarity, childhood poverty, neuroscience, neuroethics, ethics

## Abstract

Several studies have identified associations between poverty and development of self-regulation during childhood, which is broadly defined as those skills involved in cognitive, emotional, and stress self-regulation. These skills are influenced by different individual and contextual factors at multiple levels of analysis (i.e., individual, family, social, and cultural). Available evidence suggests that the influences of those biological, psychosocial, and sociocultural factors on emotional and cognitive development can vary according to the type, number, accumulation of risks, and co-occurrence of adverse circumstances that are related to poverty, the time in which these factors exert their influences, and the individual susceptibility to them. Complementary, during the past three decades, several experimental interventions that were aimed at optimizing development of self-regulation of children who live in poverty have been designed, implemented, and evaluated. Their results suggest that it is possible to optimize different aspects of cognitive performance and that it would be possible to transfer some aspects of these gains to other cognitive domains and academic achievement. We suggest that it is an important task for ethics, notably but not exclusively neuroethics, to engage in this interdisciplinary research domain to contribute analyses of key concepts, arguments, and interpretations. The specific evidence that neuroscience brings to the analyses of poverty and its implications needs to be spelled out in detail and clarified conceptually, notably in terms of causes of and attitudes toward poverty, implications of poverty for brain development, and for the possibilities to reduce and reverse these effects.

## Introduction

Contemporary neuroscientific studies of the influences of poverty on cognitive, emotional, and stress regulation systems propose to analyze how different individual and contextual factors that are associated with material, emotional, and symbolic deprivation (i.e., lack of food, shelter, education, and health-care), influence neural development. Specifically, studies in the area approach influences of developmental contexts on nervous system in terms of the analysis of neural plasticity, regulation of cognition, emotion and stress, and exposure to environmental toxins and drugs. In such a context, four central issues are: (a) the analysis of the effects of such influences at different levels of organization (i.e., molecular, systemic, cognitive, and behavioral) at different stages of development; (b) the identification of mechanisms through which these influences exert their impact (i.e., mediators and moderators); (c) how these influences are or are not modified by interventions; and (d) at what times or stages of development do such factors have the greatest impact and, accordingly, when it is more rational to implement targeted interventions to optimize self-regulation (i.e., critical and sensitive periods) (Lipina and Posner, 2012; Lipina, 2015; Lipina and Segretin, 2015; Johnson et al., 2016; Ursache and Noble, 2016).

Each of these aspects of study is associated in different ways with problems that are related to ethical implications, such as the violation of rights and dignity, decreased capacity, the determination of social responsibilities, and the potential deprivation of identity. A discussion of these topics seems to be underrepresented in the literature of neuroscience of childhood poverty and ethics. For instance, a prompt search using the keywords “brain,” “poverty,” and “ethics” results in 14 and 16 academic papers between the years 1986 and 2016 in PubMed and EBSCO, respectively. Furthermore, if the keyword “children” is added, results decrease to 4 and 5, respectively. In the same sense, the modern research area of “neuroethics” that investigates the neurobiological basis of morality and the ethical, social, and legal issues raised by neuroscientific research (Evers et al., 2017) has not begun to address these issues seriously. We suggest that filling this gap would be a valuable, interdisciplinary contribution.

Poverty is a multidimensional, relational, and dynamic phenomenon, clearly illustrated through the many conceptual definitions and indicators that disciplines such as economy, sociology, political science, epidemiology, and anthropology have generated during the last 200 years (Spicker et al., 2006). In general, there are three main approaches to consider poverty: (a) as a *material* condition in which needs, pattern of deprivations, and limited access to resources are the main components; (b) as an *economic* circumstance, in which standards of living, inequality, and the economic position are the main components; and (c) as a *social* circumstance, in which lack of basic security, exclusion, dependency, and social class are the most referred components. Furthermore, the unidimensional approaches attempt to identify how many people live in some type of poverty in terms of one indicator, or a set of indicators, that relate to an income or a non-income criterion (e.g., income-to-needs ratio, socioeconomic status) (Roosa et al., 2005; Minujin et al., 2006). In turn, multidimensional approaches simultaneously consider several indicators of basic needs and rights such as (a) health (i.e., nutrition, infant mortality), (b) education (i.e., years of education, school enrollment), and (c) standard of living (i.e., cooking fuel, sanitation, water, electricity, floor, and goods) (UNDP, 2010). The incidence of poverty using unidimensional or multidimensional measures could be significantly different.

Specifically, the findings about the influences of poverty on neurocognitive development were identified by applying three types of classic unidimensional measures: income, parental education, and occupation. However, the experience of poverty involves a set of potential mediators that shapes a virtual ecology of protective and risk factors, which involve multiple individual and contextual mediating factors at different levels of analysis (Beddington et al., 2008). This set of factors can influence cognitive development in a positive (protective) or negative (risk) way.

The contemporary literature on developmental psychology and cognitive neuroscience of poverty postulates the following as the most important protective/risk factors: (a) prenatal maternal health (i.e., nutrition, exposure to environmental toxic agents and drugs, stressors), (b) perinatal health (e.g., prematurity, birth weight), (c) quality of early attachment; (d) environmental stressors at home and schools; (e) parenting and care styles; (f) early cognitive and learning stimulation at home, care centers, and schools; (g) parental and teachers’ mental health; (h) developmental disorders; (i) family financial stress; (j) access to social security and health systems; community resources; (k) lack of social mobility; (l) social, political, and financial crisis; (m) family, social, and cultural expectations about child development (e.g., discrimination, stigmatization, exclusion); and (n) natural disasters (Bradley and Corwyn, 2002; Yoshikawa et al., 2012; Lipina, 2015; Ursache and Noble, 2016). In addition, the evidence suggests that the influences of poverty on cognitive development are a function of the accumulation of risk factors, the co-occurrence of adversities, the individual’s susceptibility to family and social environments, and the duration of the exposure to deprivations (NICHD Early Child Care Research Network, 2005; McLaughlin et al., 2014; Wagmiller, 2015). Thus, each type of deprivation may be associated with different influences on neural development (Duncan and Magnuson, 2012; Lipina, 2016).

It is also important to note that each of these factors can be present in different types of family and social environments: it is not necessarily the case, for example, that a materially wealthy family environment nourishes or stimulates its children adequately, although it has the material means to do so, or that a relatively poor environment in material terms must be inadequate for the children’s emotional development. Therefore, in this discussion we cannot generalize and speak sweepingly about “poor” versus “rich” environments; we need to be specific and say under what precise aspect we use those terms.

In this paper, we will not discuss more deeply the implications of using different indicators of poverty, because we will focus on other aspects that would contribute more directly to the dialog between neuroscience of poverty and ethics. However, using different poverty indicators is an important aspect of the research aims in this area that should be approached for its understanding of the phenomena at neural levels of analysis and its implications in the application of findings to inform interventions and policies. Therefore, we will consider poverty as (a) a complex phenomenon that result from mechanisms of inequality, and as (b) a set of deprivations that prevent satisfaction from the rights to health, housing, education, participation, and protection, as specified in the 1948 UN Declaration of Human Rights, for example.

Finally, this paper focuses on regulation of cognition, emotions, and stress to illustrate the opportunities for dialog between neuroscience of childhood poverty and ethics, notably neuroethics. The decision to select these issues does not imply that the other aspects are neither important nor potentially productive to building this dialog. However, the consideration of all the aspects of this realm would require more than one paper. In this context, the aim of this paper is to give to an audience of researchers from neuroscience and ethics an analysis of the ethical implications of the neuroscientific evidence on the influences of childhood poverty on self-regulatory development in terms of rights, dignity, capacity, and social responsibilities, so that we might foster dialog between the involved scientific disciplines.

## Evidence of Influences and Mechanisms

### Cognitive and Emotional Regulation

The study of how poverty influenced development of cognitive and emotional regulation began in the middle of the twentieth century in the context of developmental psychology and education. Currently, the high degree of interaction between developmental psychology and developmental cognitive neuroscience adds to the integration of highly productive, conceptual proposals. For this reason, what is mentioned here as a *neuroscientific approach* also includes considerations of contemporary developmental psychology.

The results that are described most commonly in early studies by developmental psychologists and educators corresponded to the behavioral level of analysis and consisted of lower scores on standardized tests that assessed motor, verbal, and executive intelligence, fewer years of completed schooling, higher incidence of learning disabilities, and school absence (Brooks-Gunn and Duncan, 1997; Bradley and Corwyn, 2002; Maholmes and King, 2012). For language development, respondents scored lower on tasks that assessed vocabulary, spontaneous speech, grammatical processing, and communication skills (Hoff, 2006).

Regarding the neuroscientific approach, different studies have generated evidence about the association between poverty and different neurocognitive systems from the first year of life at least until adolescence (Hackman and Farah, 2009; Lipina and Colombo, 2009; Hackman et al., 2010; Raizada and Kishiyama, 2010; D’Angiulli et al., 2014; Lipina, 2015; Pavlakis et al., 2015; Johnson et al., 2016; Ursache and Noble, 2016). Living in poverty can generate structural and functional changes in the nervous system relative to not living in poverty that influenced cognitive regulation through attention, inhibitory control, working memory, cognitive flexibility, self-monitoring, planning, and reasoning processing. In the following paragraphs, we include some examples of the types of outcomes that were obtained by different research teams in recent years (see Johnson et al., 2016, and Ursache and Noble, 2016 for more comprehensive reviews). In addition, these changes may be associated with differences in the processing of information that is required to regulate the course of thoughts, emotions, and learning at specific stages of development. The use of the notion “expectations” refers to the benchmarks on standardized tests, but it does not imply necessarily the norm for any culture. This is important because it is necessary to be cautious at the time of interpreting the results of studies that consider contextual and cultural aspects to avoid misconceptions and stigmatization: there is a sizable difference between considering the neural and behavioral differences due to poverty as a deficit instead of an adaptation. In ethical terms, it is important to consider if the consequences of poverty are related to circumstances in which no basic rights are satisfied (e.g., inadequate nutrition, housing, or access to education and health services).

The current exploration of the influence of poverty on neural development has begun to incorporate new approaches to identify moderation and mediation mechanisms that can affect neural activation, cognition, and behavior. For example, Lipina et al. (2013) found that in addition to maternal education and parental occupation, the availability of reading materials, daily reading to children, and the use of computers for games influenced performance in cognitive control tasks in children 5-years-old. Hackman et al. (2015) found that the potential of homes to stimulate children’s cognitive development mediated the influences of family income on the performance of tasks that demanded working memory and planning. In addition, the same researchers found that maternal sensitivity to the emotional needs of their children, mediated the association between income and the performance of tasks that demanded working memory at 1–54 months of age, in a sample of 1009 children. Quality of childcare centers can moderate the association between the levels of disorganization at home and the performance in cognitive and emotional regulation tasks for those who suffer from rural poverty (Berry et al., 2016). These results indicate the importance of considering critical, contextual issues and, thus, the need to incorporate approaches that address different levels of organization and developmental contexts (Lipina and Segretin, 2015; Lipina, 2016).

Another important aspect that influences the role of poverty on regulatory and learning systems at a behavioral level of analysis is the trajectory of the impacts or their evolution over time. One of the most important studies in this area analyzed the relationship between the duration of exposure to poverty and different regulatory and environmental factors between birth and 9 years of age. To do this, the performance of children from families who had never experienced poverty was compared with others who had experienced it between birth and age 3, at ages 4 -9, and between birth and age 9. The results showed that the group that had always experienced poverty was the one that had obtained the lowest scores in the potentiality of home environments to foster cognitive, emotional, and learning development. In turn, a mediation analysis indicated that these associations were modulated by the inadequacy of some parenting practices to identify and to regulate material, emotional, and symbolic needs of children (NICHD Early Child Care Research Network, 2005). In addition, Hackman et al. (2015) found that family income and maternal education mediated the performance in tasks that demanded planning tasks at age 7, and that income mediated the performance in a task that required working memory at age 5. Moreover, the researchers found that these differences remained constant throughout childhood, which suggested that the relationship between poverty and cognitive regulation emerged in early childhood and persisted unchanged until the end of it. The results of these studies also emphasized the importance of considering the quality of rearing environments when designing interventions that are aimed at improving the cognitive and emotional regulatory development in populations of children who were exposed to poverty.

Recently, several researchers have begun the exploration of the influences of poverty on the activation of different neural networks through the use of structural magnetic resonance imaging (MRI). For example, Rao et al. (2010) analyzed the associations of parenting practices and the level of home stimulation for learning on the neural morphology between middle childhood and adolescence. Higher scores on a scale that assessed parenting practices (i.e., HOME) were associated with higher performance on a task that demanded episodic memory processes and with smaller volumes of the hippocampus at the age 4. At the same time, they found that the association between levels of stimulation for learning at home, which was decreased in poor homes, with hippocampal volume was not verified at age 8. This evidence suggests that the quality of parenting influences the neural organization at these stages of development. Complementing this, a number of recent studies have found variations in the volumetric and cortical thickness of the hippocampus and amygdala in different populations of poor children and adolescents, and in adults who had experienced poverty as a child (e.g., Hanson et al., 2011; Jednoróg et al., 2012; Noble et al., 2012; Staff et al., 2012). In addition, several researchers found evidence of correlations between thickness, cortical surface, and connectivity of prefrontal, parietal, temporal, and occipital neural networks and levels of poverty in children, adolescents, and young adults (Chiang et al., 2011; Hanson et al., 2013; Lawson et al., 2013; Noble et al., 2015). However, changes in gray and white matter do not co-occur necessarily, as Jednoróg et al. (2012) verified in a sample of 10-year-old children with a wide range of parental socioeconomic status (SES).

Poverty may affect neural activation. Using functional magnetic resonance imaging (fMRI), several studies have found variability in the patterns of frontal and parietal-occipital activation during the solution of tasks that demanded phonological processing. This was verified in samples of children aged 5–8 years, and in adults who had reading difficulties as children and who had grown up in poverty (Shaywitz et al., 2003; Noble et al., 2006; Raizada et al., 2008). Changes also occurred in the activation of prefrontal and limbic networks during the solution of tasks that demanded stress regulation in adults who had histories of childhood poverty (Kim et al., 2013; Gianaros and Wager, 2015). Finally, the complexity of the linguistic environment when reared as a child and levels of cortisol (a hormone that is associated with the activation of the stress regulatory system) were associated with both poverty and with the activation of different prefrontal cortex areas during the performance of a learning test (Sheridan et al., 2012).

Another series of studies on how poverty influenced brain activity has used electroencephalography. Researchers found that there were differences in resting-state activation during the first year of life (Tomalski et al., 2013), in activation during the solution of tasks that demanded inhibitory control in school-aged children (Kishiyama et al., 2009), in auditory attention in preschoolers (Stevens et al., 2009) and school-aged children (D’Angiulli et al., 2008), and in emotional processing during adolescence (Tomarken et al., 2004).

At a behavioral level of analysis, the influences of poverty on cognitive and emotional regulation and language development were mediated by the quantity and quality of home stimulation of cognition and learning, and by the language environment during the early stages of development (Lipina and Colombo, 2009; Hackman et al., 2010; Lipina and Posner, 2012; Ursache and Noble, 2016). In the last 2 years, several studies added evidence that supported this hypothesis at a neural level of analysis. For instance, the volume of gray matter and cortical thickness in frontal and temporal areas were identified as mediators of the association between income and academic performance at ages 4–18 (Hair et al., 2015). The connectivity between the hippocampus and the amygdala was identified as a mediator of the association between income and symptoms of depression during the preschool stage, in poor children ages 7–12 (Barch et al., 2016). In addition, the connectivity between different neural networks that involved several cortical areas was identified as a mediator between the number of years of education and performance on cognitive control tasks during adolescence (Noble et al., 2013). The structural neural mediation between functional and environmental phenomena is still an issue of debate that should be analyzed more carefully, because the dynamics of the relationships between events that are evident at different levels of organization are still not well understood. Consequently, a hurried use of these findings has the potential to encourage more research and replication to substantiate these hypotheses (e.g., that the biological level of analysis determines the relationships between the behavioral and environmental levels).

### Stress Regulation

Since the mid-20th century, several studies have analyzed the regulatory stress response in children and adults as one of the most important mediating mechanisms of the influence of poverty on cognitive, emotional, and social functioning (Doom and Gunnar, 2013). Threats, negative events, exposure to environmental hazards, family and community violence, changes in the dynamics of family life, job loss, instability, and economic deprivation, which are more likely to occur under poverty conditions (Evans, 2004), are all phenomena that have the potential to activate different systems of stress regulation (Bradley and Corwyn, 2002; Maholmes and King, 2012; McEwen et al., 2015). The physiological stress responses can be manifested, for example, through vagal tone^1^, allostatic load, and neuroendocrine activity^2^. One of the main neural systems that is associated with the implementation of this complex regulation includes the pituitary, hippocampus, amygdala (i.e., HPA axis), and different areas of the prefrontal cortex. The HPA axis begins to respond to stress signals during the prenatal stage. Currently, there is evidence that this information can produce physiological and epigenetic changes with possible long-term consequences on physical and mental health (Christian, 2015). After birth, the HPA axis continues its development and expresses high levels of reactivity during the first months of life. One of the most important consequences of this level of immaturity and high reactivity is that during the first 3 months of life, any variation in the care of children is reflected in the activity of the HPA axis. Precisely, this is an important period, because of the establishment of emotional attachments between caregivers and children. What neuroscientific research should elucidate in future studies is whether this degree of response implies the existence of a critical or sensitive period^3^ during which normal variations in childcare could program the functioning of the HPA axis in later stages of development.

Recent evidence suggests that the experiences of abandonment during the first year of life may be associated with persistent changes in neural structures that are associated with volumetric changes in the hippocampus (Hodel et al., 2015). At approximately the fifth month, the HPA axis begins to stabilize its regulation and to be less reactive to subtle changes in parenting practices. From that moment, it begins a stage in which it is more difficult to verify increases in the release of cortisol; that is, this occurs at a stage of development in which children would be protected by secure attachments with their caregivers. It is difficult to determine how early it is possible to identify this attachment damping effect on the functioning of the HPA axis and the release of cortisol, which is the underlying mechanism, and how early it would involve neural networks in the hypothalamus and the prefrontal cortex, and the oxytocin and opioid systems (Doom and Gunnar, 2013).

Although the prenatal and early childhood stages of development could be sensitive periods for the development of the stress regulation systems, neuroscientific evidence suggests that they are not the only sensitive stages, because increases in the production of cortisol are also evident during adolescence. In turn, these changes in the HPA axis could increase the neural vulnerability to stress during early adolescence, which would explain, in part, the regulatory changes to cognitive and emotional levels that occur during this stage (Doom and Gunnar, 2013). The experience of environmental adversity during puberty, such as maltreatment, could be associated also with changes in the volume of structures that are associated with the HPA axis (e.g., amygdala) during adulthood (Pechtel et al., 2014).

During these stages of development, stress and uncertainty that are generated by the conditions of economic deprivation increase the likelihood of occurrence of negative emotional states, anxiety, depression, and anger. In turn, such emotional conditions can induce a higher frequency of negative, parental control strategies, less emotional sensitivity to children during development, and greater difficulties in implementing appropriate parenting practices that are aimed at fostering cognitive and emotional regulatory development (Shonkoff et al., 2012). However, even in poverty, maintaining proper parenting practices can result in a protective factor (Brody et al., 2002), which highlights the importance of the influence of interventions on children’s regulation systems during development.

Current neuroscientific studies in this area have begun to incorporate the concepts and methodologies that are derived from advances in epigenetics and the analysis of neural activation (Evers and Changeux, 2016). In particular, there are three issues that feed the current studies in the area: prenatal programming, reactivity of the amygdala to threatening experiences, and the embodiment of adversities (Gianaros and Manuck, 2010). For example, in the study of the long-term consequences of stress experiences in child poverty, Blair et al. (2011) found that cortisol levels, in combination with parenting practices, were mediators of the association between poverty and the performance of tasks that tapped self-regulatory demands. These results suggested that childhood poverty and maternal mental health modulated the regulation of stress, which are two factors that could improve our understanding of the associations between childhood poverty and stress regulation. Specifically, the experiences of physical and sexual abuse during early stages of development have been associated with a complex pattern of responses to stress, which are assumed mediators of increased susceptibility to the development of psychiatric disorders in adulthood (Feder et al., 2009). One hypothesis is that epigenetic changes in the encoding of glucocorticoid receptors mediates the association between stress and cognitive and emotional regulation in adolescents with a history of child abuse (Romens et al., 2015). However, vulnerability and susceptibility to situations of moderate stress vary among individuals according to different epigenetic mechanisms and the possible presence of certain protective factors, such as relationships with sensitive adults (Shonkoff et al., 2012; Evans and Fuller-Rowell, 2013).

Finally, during the last decade, the first neuroimaging studies have begun to explore how socioeconomic deprivation during childhood influences the response to stress in different stages of life. For instance, Tottenham et al. (2011) evaluated the long-term impact of adverse conditions during childhood on adult performance in tasks that required emotional processing of threatening faces. The results of this study showed that amygdala reactivity increased in children who were raised in orphanages, probably because they had decreased visual contact with adults during interactions.

## Reducing the Negative Impacts of Poverty

Since the mid-20th century, several researchers have begun to design and implement different intervention programs to reduce the negative impacts of poverty on cognitive and emotional regulation. Such efforts have emerged simultaneously within different humanities, social, and health sciences. The basic concept of this type of intervention program is that given the multidimensional nature of the phenomena of childhood poverty and development, any intervention that is aimed at optimizing the conditions and opportunities for development of children who live in poverty require the same type of complexity. This challenge involves designing multiple intervention modules that incorporate actions for children, families, teachers, civil organizations, and governments, and developing the genuine integration of different conceptual and methodological perspectives. In the fields of developmental psychology, education, nursery, and social work, most of these actions were designed in the form of programs that offered activities and services for children, families, schools, and communities. In many of these cases, interventions only consisted of the implementation of a single module of intervention that was oriented only to one aspect (e.g., cognitive training). But in other cases, programs have been designed and implemented, which included several activities that addressed simultaneously different dimensions of the phenomenon of poverty, and which provided a set of articulated actions that were implemented in different developmental contexts (National Research Council, and Institute of Medicine, 2009; Burger, 2010; Barnett, 2011; Sandler et al., 2011; Yoshikawa et al., 2012; Barry et al., 2013; Blair and Raver, 2014; Slopen et al., 2014; Fiorella et al., 2016; Fisher et al., 2016).

During the last decade and a half, some researchers have developed interventions that were also aimed at improving children’s cognitive development using concepts and methodologies that stemmed from recent developments in cognitive neuroscience. The main aim of these interventions was to analyze the plasticity of several neurocognitive systems through the exercise or training of basic cognitive processes. These interventions have been performed in samples of children with no history of disorders (e.g., Rueda et al., 2005; Stevens et al., 2009; Thorell et al., 2009), or with children who had reading, arithmetic, and ADHD disorders (McCandliss et al., 2003; Temple et al., 2003; Klingberg et al., 2005; Wilson et al., 2006), or with children who were living in diverse SES homes (e.g., Colombo and Lipina, 2005; Diamond et al., 2007; Neville et al., 2007; Stevens et al., 2009; Wilson et al., 2009; Goldin et al., 2014; Segretin et al., 2014; Hermida et al., 2015; Ballieux et al., 2016).

In general, with limited exceptions, such interventions do not consider the ecological and systemic approaches to child development. For this reason, there is still a need to explore the possibility of articulating the approaches of multimodular intervention and cognitive neuroscience.

Some examples of interventions designed in the realm of developmental psychology and cognitive neuroscience implemented in Argentina and the USA, are presented here. The School Intervention Program was implemented in the city of Buenos Aires (Argentina) during 2002-2005, with the aim of optimizing the development of executive function of preschoolers who were living in poverty. The results indicated that exposure to a module of individual cognitive exercise plus a nutritional supplement of iron and folic acid was the most effective way to improve the level of performance in tasks that demanded attention, working memory, and planning processing in children who were 4–6 years old (Colombo and Lipina, 2005; Segretin et al., 2014). Another intervention implemented by the same research team was the Curricular Intervention Program had the aim of optimizing performance of executive function of children who were living in poverty through school activities adapted to the educational curricula of the city of Buenos Aires, and this was administered by teachers. Results of cognitive assessments showed only an attentional improvement in the intervention group. However, when analyzing the grades assigned by teachers during the first grade of primary school, a year later, children in the intervention group scored higher in math, language, and behavior (Hermida et al., 2015). A third intervention designed by the same group in collaboration with researchers from the Laboratorio de Neurociencia Integrativa (LNI) at the University of Buenos Aires called *Mate Marote*, consisted of the administration of computerized games that were aimed at training different cognitive control processes (intervention group) in comparison with commercial games with lower cognitive demands (control group). The results indicated significant increases in tasks that required attention, inhibitory control, and fluid processing, and better grades in language and mathematics in those children with higher rates of school absence (Goldin et al., 2014).

The PCMC-A project by Neville et al. (2013) implemented two modules with children and their families from poor homes in Eugene, Oregon. One of the modules consisted of attentional training activities for children; the other module involved weekly meetings (*n* = 8) with families, during which researchers systematically approached and discussed with parents several aspects of parenting and family communication practices. The results showed improvements in cognitive, behavioral, and electrophysiological measures, which indicated attentional, language, and behavioral improvements in children, and a decrease in the perception of parental stress with concomitant improvement in family communication. Recently, Ballieux et al. (2016) implemented computerized attentional control training at a child-care center to a sample of 12-month-old infants from low- and middle-SES homes throughout 5 weekly sessions. Results showed training-related improvements on tasks that involved visual sustained attention, saccadic reaction time, and rule learning.

Overall, performance on tasks that demands cognitive and emotional regulation of children who live in poverty can be optimized through innovative interventions in the laboratory, child-care centers, schools, and even in homes. Thus, this type of training has the potential to be implemented in different developmental settings and to help reduce some impacts of poverty on self-regulatory development (Blair and Raver, 2014). The reduction and the potential immutability of the negative impacts are different phenomena that should be analyzed with distinct neural underlying mechanisms in mind.

For instance, based on experiments that were implemented in animal models, the reduction of the impacts on the nervous system through environmental enrichment were related to changes in synaptic plasticity during the life cycle (e.g., Morley-Fletcher et al., 2003; Winocur et al., 2005; Holtmaat and Svodboda, 2009; Hirase and Shnohara, 2014). Irreversibility was specifically related to changes in the functional maturation of local inhibitory connections of competing sensory inputs, which in turn was mediated by reorganization of the extracellular matrix, all of which occurred during critical periods (Hensch, 2005). In other words, reduction was more related to dependent-experience plasticity and irreversibility was more related to expectant-experience plasticity, which also implied that different temporal scales and dynamics were involved in each type of plastic process (Murray et al., 2014). The elucidation of mechanisms of reversibility or irreversibility of impacts of childhood poverty on self-regulation still depends on the implementation of innovative designs that allow the exploration of these plastic mechanisms at different levels of neural organization. In summary, the evidence of the intervention studies implemented with children from poor or low-SES backgrounds supports the notions that (1) poverty does not necessarily imply immutability of their impacts on the development of cognitive and emotional regulation (Lipina, 2015), and (2) there is replicated evidence that suggests what aspects of design of interventions increases their potential efficacy (e.g., Ramey and Ramey, 1998; Barnett, 2011). However, it is necessary to explore and to improve several conceptual and methodological issues, such as size effects (usually low or moderate), the transfer of cognitive gains to other domains of development, and the variability of results that depend on epigenetics (Obradović and Boyce, 2009), temperament (e.g., Vijayakumara et al., 2014), cortisol response (e.g., Dadds et al., 2015), motivation, preexisting ability, and implicit theories of intelligence (Jaeggi et al., 2013; Clark et al., 2016; Katz et al., 2016).

## Scope and Limits of the Available Evidence

The neuroscientific study of how poverty influences cognitive and emotional regulatory development is still at an early stage. However, after a decade of applying neuroimaging techniques and paradigms of neurocognitive functioning, we can state that: (1) the different experiences of adversity related to poverty are associated with changes in the structure and function of neural systems that are related to cognitive and emotional regulation, language, and learning skills; (2) these influences could occur at different times during human development; (3) the hypothetical mechanisms through which these changes occur involve different factors that are related to childcare through the quality of language environments, and through cognitive stimulation and emotional support at home and in educational contexts.

However, as in any scientific endeavor, there are aspects of this topic that need deeper understanding to avoid generating misconceptions that may contaminate the social uses of this knowledge. One such aspect is the interpretation of the results that are obtained by applying different neuroimaging techniques. For instance, a pattern of electrophysiological activity that indicates differences in attentional processing from what would be expected for children of the same age who do not live in poverty (e.g., Stevens et al., 2009) does not necessarily mean that such patterns represent a dysfunction or a deficit. Actually, this kind of evidence suggests that we are facing an adaptive process, which is also possible to modify by intervention (Neville et al., 2013), so it is necessary to improve our understanding of the contextual conditions that are associated with these adaptations. This could stimulate genuine interdisciplinary efforts to integrate concepts of ecological psychology (e.g., Barker and Wright, 1948; Bronfenbrenner, 1979). In any case, it is not appropriate to communicate that such findings correspond to an immutable and irreversible deficit condition. In the case of using techniques of neural structural analysis, this issue must be analyzed much more carefully, because the information is not obtained by analyzing the concomitant functional activity, as in the case of EEG and fMRI techniques. However, it is possible to verify examples of social communication of these results that are problematic, because they induce, explicitly or implicitly (e.g., Reardon, 2015), false notions that poverty generates immutable impacts.

For example, in 2013, a note published on the Smithsonian’s website stated that growing up in poverty can affect brain development, and that a large body of research shows that circumstances of poverty and chronic stress disrupt brain development (Stromberg, 2013). In 2014, the newspaper The Guardian published a note stating that half of Afghan children suffer from irreversible damage from malnutrition, and that poor nutrition in the first 2 years has permanent effects on growth and development (Graham-Harrison, 2014). And, the following statement is on the UNICEF-China website: “*Children are more vulnerable to poverty than other age groups. They experience poverty differently to adults/other household members and their needs vary at different ages. Investing during critical periods, particularly in early childhood, is crucial to combat child poverty. Time-sensitive processes of development/maturation mean that the outcomes of child poverty are profound, long lasting and irreversible.*” In 2015, researchers from nine American research centers published an article in the journal Nature Neuroscience (Noble et al., 2015), in which they showed new evidence on the influence of childhood poverty on brain structure and cognitive performance. They claimed that it was not possible to interpret these results as meaning that childhood poverty had irreversible effects. However, the same week the magazine Nature (from the same publishing company) published a news note entitled “*Poverty shrinks brains from birth.*” A few weeks ago, the Interamerican Development Bank blog provided a set of similar considerations about early childhood and brain development: “*It is in the first 3 years of life that the human brain grows more than at any other stage, 80% of the adult size, and this is why learning is performed more easily than at any other time. During this short but unique period, children need adequate attention, stimuli and interactions that allow them to develop their greatest potential at the cognitive and non-cognitive levels. Some deficits in appropriate stimuli during early childhood can be compensated for later, but the cost is so high that the damages are often irreversible.*”

It is also important to advance the analysis of causality in this area, and to avoid communicating the correlational evidence as if it were causal. In this regard, it is necessary to advance the design of longitudinal studies that involve different levels of organization and multiple mediation analysis to allow: (1) the identification of the differential effects of the accumulation and/or combination of several types of adversities throughout development; (2) the understanding about how different types of adversities modulate the efficiency of distinct neural networks; (3) the identification of periods of vulnerability and greater sensitivity to different types of adverse experiences; (4) the exploration of phenomena of immutability; and (5) the design of valid measures to assess cognitive and emotional regulation in different stages of development.

Available evidence from intervention studies indicates that the influences of poverty on the structure and function of the nervous system are not necessarily irreversible and immutable. However, this area is at an early stage and needs to integrate ecological conceptual approaches. Finally, it is also necessary to explore the design and implementation of studies that address individual and environmental differences to allow the expansion of their potential benefits to different subgroups of participants.

## Neuroethical Implications

Research on poverty gives rise to numerous important ethical and social-political issues. There is an extensive literature on the mechanisms of poverty in social science, which include ethical perspectives (Hunt and Bullock, 2016), and it is beyond the scope of this paper to discuss all. Here, we wish to draw attention to neuroethical perspectives that we consider being fundamental, and which suggest further studies. Specifically, we should stress the relevance of neuroscientific evidence about poverty in arguments for reducing its impacts, and the importance of communicating these arguments in a way that does not stigmatize individuals or social environments (e.g., through unjustified generalizations).

The rights to adequate nourishment, housing, education, and health care are listed in the UN General Assembly (1948, §§25–26). These rights are contested in different political systems and cultural contexts where there is a tendency to deny these rights as a social responsibility and, instead, to explain poverty as a personal failure of the person aﬄicted (e.g., Feagin, 1972, 1975, and the review by Hunt and Bullock, 2016). In other words, poverty is not regarded universally as a consequence of unequal access to social benefits. The latter, individualistic views are common in North America and South America, where the problem of poverty is immense compared to western and northern Europe, where social views on poverty are more dominant. It is worth noting that countries and political systems that accept the above-mentioned rights as a shared social responsibility are also among those who have been most successful in combatting poverty, social violence, and insecurity [e.g., the Scandinavian countries (cf. Eurostat, or the World Bank Global Poverty Overview)].

We shall not enter this last discussion here except by pointing out that the evidence from neuroscience renders the individualistic way to explain poverty as a personal failure is, to some extent, absurd. For example, an infant does not choose into which social context it is to be born, and when its brain is prevented from developing in a healthy manner due to poverty and its surrounding social conditions (lack of adequate nourishment, housing, nurseries, schooling, health care, etc.), then it is certainly not a question of personal failure. In addition, the parents cannot be held entirely responsible for a socio-political context that does not provide the means for offering each child a healthy development, unless one is prepared to argue that poor people should not have children; with such a conclusion, one deserts the discourse of democracy altogether.^4^ Evidence from neuroscience goes to show not only how poverty may breach human rights, but also how it may prevent a child’s possibility of ever enjoying them. In other words, it importantly reveals how poverty may cause problems in the very prerequisites for attaining personal development and a good life.

From an ethical perspective, perhaps the two most important questions to arise from this research are: (1) how can these results be useful in the social quest to diminish the extent of poverty (when poverty remains present)?, and (2) how can these results help diminish the negative effects of poverty? These questions suggest implicitly that poverty should be diminished. Politically, this is not a globally endorsed position, as statistics on poverty reveal. *Homo sapiens* is a beast of prey, sharing does not come naturally, and sharing equitably does not come at all, with few exceptions. We are profoundly hierarchical and selective in our attitudes toward distributing benefits (Evers, 2010, 2016) and even though, materially, poverty could be abolished theoretically, it remains present in most societies, including wealthy welfare states (Bradshaw et al., 2007). The point here is that the desire to abolish or even diminish poverty cannot be taken for granted in the populations at large or among the politicians governing them. Therefore, important and noble as the human rights discourse is, it may not be sufficient to argue for the reduction of poverty simply because of new neuroscientific knowledge about its detrimental effects on the developing brain, with reference to human rights, or human dignity. Appeals to empathy, goodwill, or social conscience risk convincing only those who already endorse such views, and leave the rest untouched (who may well be in the majority).

A complementary way to argue for the reduction of poverty in contemporary societies is by referring to the self-interest of those who are not poor (i.e., to show that it serves their interest to have less poverty around them). This is not a new approach. It has been tried before in attempts to explain how egalitarian health-care is cost-efficient, quite independently of goodwill or other humane concerns (Evers, 1997), or that problems of social violence and insecurity are not best solved by increased state-violence, but by reducing poverty in early childhood (Heckman, 2006). So far, the results are disheartening. For example, after a decade of modest improvements in life-conditions, childhood poverty in Argentina has again risen dramatically (Tuñón, 2016), and the ideal of public, free schooling for all children still remains a distant dream in many countries, in spite of the positive outcomes of introducing education in poorer areas such as favelas in Brazil. Clearly, even an appeal to rational self-interest may fail, if inequality is endorsed as a value that supersedes self-interest. For instance, this might be true if the belief that one belongs to a small, select “elite” is connected to a satisfaction that outweighs other values, such as security.

Nevertheless, the knowledge that neuroscience can bring to this issue, jointly with knowledge from the social sciences and humanities, provides considerable support for the idea that reduction of poverty benefits society at large, and that this argument must be pursued. It is an important task for neuroethics to engage in this interdisciplinary research domain to contribute analyses of key concepts, arguments, and interpretations. The specific evidence that neuroscience brings to analyses of poverty and its implications, needs to be spelled out in detail and clarified conceptually. Notably:

1. *Causes of poverty and attitudes to poverty*. For example, comparing individualistic versus systemic or social explanations, what does neuroscience concretely contribute to these debates? On the other hand, considering the neural and behavioral differences due to poverty as a deficit instead of adaptations – what does this imply?
2. *Impacts of poverty on brain development.* The interpretation of evidence and the identification of ethical and social issues that arise might provide the means for reducing poverty’s negative impacts.
3. *Questions of immutability*. Which impacts of poverty can be changed, and how? What does the concept of “reversibility” entail? The evidence available in this area raises specific ethical challenges that should also be considered in the interpretation of results and the planning of future research^5^.

One such challenge involves the need to avoid misconceptions, and the potential deprivation of identity for those living in poverty, through the social communication that disseminates notions of irreversibility or mental weakness just because one is poor. To the extent that irreversible damages in the form of mental problems or disorders result from poverty, these results must be communicated in a way that avoids stigmatization or misuse (e.g., in terms of over-interpretations, or extrapolations). Therefore, it is important to analyze carefully the scope of the results, and to consider the complexity of the development of cognitive and emotional regulation, and the characteristics of the cultural contexts.

We have suggested above that neuroscientific evidence is relevant both to reduce the negative impacts of poverty and to reverse the impacts once present, but we also pointed to some risks that need to be considered. Primarily, there is a risk of stigmatization of people (individuals or populations) living in poverty. How to communicate research without unjustified generalizations, or over-interpretations, is a special challenge in an area of research that is permeated with political and social ideologies. Furthermore, as the neural costs of poverty imply, the reduction of capabilities and rights include increased morbidity and premature mortality from preventable causes throughout the life cycle. Preliminary evidence indicates that interventions can reduce such costs, so it is necessary to question our responsibility regarding the suffering of those among us who are experiencing poverty.

Finally, we propose a series of recommendations that could contribute to deepening the interdisciplinary dialogue between neuroscience and ethics:

(a) To generate debates between researchers in neuroscience, social science, ethics and neuroethics, journalists, and policymakers so that they base social communication and policy design on the limits imposed by the evidence, the consideration of children’s rights, and the dignity of those who suffer from poverty. In particular, the design of policies and the ethical implications they entail could be included in the curricula during the degree and postgraduate courses of the involved disciplines. In particular, in this context of integration, it is important to discuss the ethical implications of the manipulation of critical periods.
(b) To promote the theoretical integration of neuroscientific findings with concepts of cognitive development theories, as was proposed by Crone and Ridderinkhof (2011), to include ecological multilevel approaches, and to overcome misconceptions about brain structure and function development.
(c) To foster interdisciplinary efforts aimed at improving the design of interventions by exploring the potential usefulness of integrating the neuroscientific approach to multimodular interventions, and to foster the design of experiments aimed at exploring the modifiability of the opening and closing of critical periods.
(d) To incorporate measures of individual differences into impacts and intervention studies to improve effect size and transferability, and to identify indicators of potential usefulness for policy surveys.

## Author Contributions

Both authors contributed equally to the selection of key questions and content, as well as with the discussion.

## Conflict of Interest Statement

The authors declare that the research was conducted in the absence of any commercial or financial relationships that could be construed as a potential conflict of interest.

## Footnotes

1 *Vagal tone* refers to the biological activity of the vagus (tenth cranial) nerve, which originates in the medulla oblongata of the brainstem. It is a key component of the autonomic nervous system, which regulates the resting state of the majority of several organ systems, such as the heart, lungs, eyes, glands, and digestive tract. In the context of psychophysiological research, vagal tone represents an index for the functional state of the entire parasympathetic nervous system.

2 Adaptation to stressful circumstances involves the activation of several neural, neuroendocrine, and immune mechanisms. This adaptation is called *allostasis*, which is an essential component of maintaining homeostasis. Stress hormones in combination with other physiological processes (e.g., increased myocardial workload, decreased smooth muscle tone in the gastrointestinal tract) have protective and adaptive effects in the short term. However, they can also increase pathophysiological processes when they are overproduced or mismanaged. Constant or even irregular exposure to these hormones can eventually induce illnesses and weaken the activity of the immune system. Over longer time intervals, the resulting *allostatic load* exacts a cost that can accelerate disease processes. Allostatic load can be measured in physiological systems as chemical imbalances in autonomic nervous system, central nervous system, neuroendocrine, and immune system activity as well as perturbations in the diurnal rhythms and, in some cases, plasticity changes to brain structures (McEwen, 2000).

3 In developmental psychology and developmental biology, a *critical period* is a maturational stage in the lifespan of an organism during which the nervous system is especially sensitive to certain environmental stimuli. If the organism does not receive the appropriate stimulus during this period it may be difficult, ultimately less successful, or even impossible, to develop some functions later in life. Functions that are indispensable to an organism’s survival at early stages of development, such as sensory systems (e.g., vision, hearing) and attachment are particularly likely to develop during critical periods. Some researchers differentiate between *critical* and *sensitive*, defining the latter as a more extended period, after which learning is still possible, as in the case of cognitive self-regulation skills, which are based in the integration of maturational plasticity of different neural networks during the first two decades of life (Romeo, 2014).

4 In fact, this would be the spirit of the English economist David Ricardo (1772–1823), an icon of political liberalism, who suggested that wages should be measured by subsistence level, which would allow workers barely to survive, while preventing them from reproducing freely, an idea that was favorably taken up by the philosopher Sidgwick.

5 In this section, we do not approach those issues of research procedures that raise ethical implications regarding potential difficulties in the asymmetry between researchers and subjects, excessive use of technical jargon during interviews, the need to consider the eventual traumatic situations during interviews, and eventual difficulties regarding subjects’ consents and assurance of voluntariness to withdraw from the studies.
